# Mutation and Recombination Rates Vary Across Bacterial Chromosome

**DOI:** 10.3390/microorganisms8010025

**Published:** 2019-12-21

**Authors:** Maia Kivisaar

**Affiliations:** Chair of Genetics, Institute of Molecular and Cell Biology, University of Tartu, 23 Riia Street, 51010 Tartu, Estonia; maia.kivisaar@ut.ee

**Keywords:** mutation rate, homologous recombination, mutation accumulation, bacterial chromosome structure, nucleoid-associated proteins, evolution

## Abstract

Bacteria evolve as a result of mutations and acquisition of foreign DNA by recombination processes. A growing body of evidence suggests that mutation and recombination rates are not constant across the bacterial chromosome. Bacterial chromosomal DNA is organized into a compact nucleoid structure which is established by binding of the nucleoid-associated proteins (NAPs) and other proteins. This review gives an overview of recent findings indicating that the mutagenic and recombination processes in bacteria vary at different chromosomal positions. Involvement of NAPs and other possible mechanisms in these regional differences are discussed. Variations in mutation and recombination rates across the bacterial chromosome may have implications in the evolution of bacteria.

## 1. Introduction

Several studies have suggested that mutation and recombination rates are not constant across the bacterial chromosome. Mutation rate can vary depending on the local context of the DNA sequence. In addition, other factors such as the level of transcription of the target gene, DNA strand bias, and transcription and replication machinery collisions affect mutation rate [1,2,3,4,5,6,7,8,9,10]. Also, host factors affecting DNA topology may influence mutagenesis and recombination processes both on a local and on a larger scale. Bacterial chromosomal DNA is folded into a complex structure called the nucleoid. Based on chromosomal conformation capture and DNA–DNA interaction studies, the nucleoid is divided into dynamically distinct domains called macrodomains: Ori (including the origin of replication *oriC*), Left, Right, Ter (including the terminus of replication, *ter*), and two unstructured regions that flank Ori [11,12]. The organization of the chromosome into macrodomains was discovered by analyzing the frequency of site-specific recombination between pairs of sites scattered over the chromosome. This analysis demonstrated that there are regions with high internal frequency of recombination and that DNA interactions between different macrodomains are highly restricted [11]. The level of DNA supercoiling in nucleoid is tightly regulated by the combined activities of topoisomerases and nucleoid-associated proteins (NAPs) such as Fis, HU, H-NS and IHF [13,14]. The abundance of NAPs in the bacteria changes during the transition from exponential growth phase to stationary growth phase [15,16]. These proteins can induce dynamic alterations in the physical structure of the promoters in the bacterial nucleoid and therefore directly affect the patterns of gene regulation [17,18]. NAPs bend, wrap or bridge DNA, having different affinities to DNA, and they have recently been shown to be involved in the higher-order chromosome organization as well [12]. It has been shown that changes in the activity of NAPs influence DNA replication and repair and transposition of mobile DNA elements [19,20,21]. In addition, gratuitous repair could also be stimulated by specific DNA structures or dynamics such as transcription [22]. The reader can find detailed overviews on the structure of the bacterial chromosome and on the functions of NAPs in genome organization and activity in several recent reviews, e.g., [23,24,25,26,27]. The aim of the current review is to give an overview of recent observations regarding the effect of target sequence location in the bacterial chromosome on mutagenesis and recombination processes, as well as to describe the role of NAPs in these processes.

## 2. Mutational Processes Vary Across the Bacterial Genome

It is commonly known that some variation in mutation rates exists at the gene level [28,29,30]. A growing body of evidence has accumulated showing that the mutation rates vary also at a larger scale, between different chromosomal regions. Earlier studies on genomic data analysis indicated that the genes located further from the replication origin have higher mutation rates than those nearer to it [31,32]. Some experimental studies that have compared mutation rates in a reporter sequence inserted at different locations in the bacterial chromosome have also reported on the impact of the chromosomal position of the target sequence. However, these studies did not find a correlation between the increased mutation rate and the distance from the origin of replication [10,33,34]. For example, the reversion rate of *lacZ* alleles inserted at four positions in the *Salmonella enterica* chromosome revealed that mutation rates at an intermediate locus were significantly higher than those at loci nearer to and farther from the replication origin [33]. Martina and colleagues [34] examined the effect of chromosomal context on frameshift mutation rate in *E. coli* wild-type and *mutS*-deficient strains carrying a mutant chloramphenicol acetyl transferase gene allele at different locations in the chromosome. They observed that chromosomal context affects both the fidelity of DNA replication and the efficiency of mismatch repair. In *Pseudomonas putida* the variation in the rate of mutations at different chromosomal positions indicated a complexity of mechanisms that could affect mutagenic processes [10]. In this study, the test systems either detecting mutations that inactivate the *lacI* repressor or frameshift mutations within a run of seven C-nucleotides in the phenol monooxygenase gene *pheA* were inserted randomly into *P. putida* chromosome. Mutation rate was elevated when the direction of transcription of the mutational target gene was opposite to the movement of replisome, and the higher level of transcription of the tester gene also facilitated occurrence of mutations. In addition, the frequency of frameshift mutations was elevated when the tester gene *pheA* was oriented so that the template for the leading strand synthesis contained the C-nucleotide run [10].

It should be noted that the discussed in vivo assays discussed above allow to compare only relatively a few chromosomal locations. Comprehensive study of the variation in mutation rates in bacterial chromosome has been made possible only recently with contemporary DNA sequencing methods and a sharp decrease in whole genome resequencing cost. A powerful approach in studying mechanisms affecting mutation rates in bacterial chromosome is to use mutation accumulation (MA) experiments followed by whole-genome sequencing (WGS) of MA lines. The concept of MA studies was first applied for mutagenesis experiments with eukaryotic organisms, and it was meant to allow accumulation of spontaneous mutations under conditions of minimal selection in initially homozygous lines of very small effective size [35]. MA experiments in prokaryotes typically involve picking and streaking single colonies on agar, whereas each passage is followed by approximately 25–30 generations of growth. A single clonal ancestor is used to initiate many replicate lineages, and the population of cells in the colony is passed through hundreds of single cell bottlenecks before each lineage is subjected to WGS [36,37,38]. Multiple lines of evidence suggest that selection is negligible in MA studies of prokaryotes, and the rates and patterns of mutations in prokaryotic genomes have not been biased by selection during repeated colony re-streaking [37,38,39]. Therefore, the MA-WGS studies should yield a highly accurate estimate of the spontaneous mutation rate of bacteria. For example, the estimation of total genomic mutation rate to 3.4 × 10^−3^ per genome per generation of *Salmonella typhimurium* [40] was very close to that determined by Drake [41] on *lacI* and *his* operon in *E. coli*. However, in the case of *E. coli*, the estimates obtained from MA-WGS studies were threefold lower than Drakes’ value measured on specific loci [38,41]. Moreover, the rate of base substitution mutations from MA-WGS experiment was six- to nine-fold higher than that obtained from the classical fluctuation tests scoring for resistance to rifampicin or nalidixic acid [38]. The authors explained this discrepancy by phenotypic lag required for antibiotic-sensitive molecules to be replaced by resistant ones before the resistance phenotypes are expressed in the newly arisen mutants. Nevertheless, single-locus studies are still necessary for our understanding of mutational processes because, for example, WGS does not identify true mutational hot spots [38].

MA-WGS experiments are usually focused on the base substitutions, because these types of mutations occur at 10-times the rate of indels in wild-type cells, and five-times the rate of mutations in mismatch repair-deficient cells [38,42]. MA-WGS experiments with mismatch-repair defective *E. coli* revealed that the identified base substitutions fall into wave-like patterns of increasing and decreasing mutational densities that are mirrored in the two separately replicated halves of the bacterial chromosome [37,43]. The similar wave-like pattern of the base substitution rates across chromosome became evident also in other bacterial species such as *Pseudomonas fluorescens* [44], *Pseudomonas aeruginosa* [45], *Bacillus subtilis* [43], *Vibrio cholerae* [46,47] and *Vibrio fischeri* [46]. The effects of genomic position on mutation rates can be studied in much greater detail in mismatch repair-deficient (MMR^–^) lineages where mutations are distributed across the genome at shorter intervals than in wild-type strains. Therefore, most of the MA-WGS experiments have been performed with MMR-deficient bacteria. However, a wave-like pattern similar to that of *P. aeruginosa* wild-type and MMR^–^ MA lineages, with peaks in the distribution near the terminus (2.75–3.0 Mb), and intermediate between the origin and terminus (1.25–1.50 Mb), was observed also in genomic analysis of clinical isolates of *P. aeruginosa*, suggesting that the mutation distribution bias during DNA replication may affect the levels of diversity observed along the chromosome in natural populations [45].

What are the mechanisms causing the wave-like pattern of mutations across the bacterial chromosome? Dillon and colleagues [48] have tied periodic variation of mutation rates with replication timing. The genomes of *Vibrio cholera* and *Vibrio fischeri* are composed of two circular chromosomes and the genome of *Burkholderia cenocepacia* is composed of three. In these bacteria the replication initiation from the first and the largest chromosome starts earlier to ensure that replication of all the chromosomes terminate synchronously [49,50,51]. Such a replication timing mechanism has enabled to examine whether secondary chromosomes have similar mutation rates and regional variation to concurrently late-replicated regions of the primary chromosome. Dillon and colleagues found that the base substitution rates in the secondary chromosome were similar to those in the simultaneously replicated regions of the larger chromosome, suggesting that the periodic variation in mutation rates across bacterial chromosomes is associated with replication timing [48]. Based on the results of this study [48] and the earlier finding that the high levels of dNTPs elevates mutation rate in *E. coli* [52], Dillon and colleagues proposed a model that mutation rates vary in concert with fluctuations in dNTP levels when the replication is initiated repeatedly during the rapid growth of the bacteria. As the replication fork progresses, the dNTP levels decline, but during the exponential growth phase of the bacteria each replication initiation creates another burst of dNTP synthesis. The experiments performed in P.L. Foster laboratory [43,53] have provided a partial support for this hypothesis. These studies have demonstrated that the increased levels of dNTP elevate mutation rate and shift the mutation rate peak away from the *oriC* [43]. The reduced growth of *E. coli* due to cultivating bacteria on glucose minimal plates instead of rich growth medium (on LB plates) or on LB plates at lower temperature (at 28 °C) reduced the rate of base substitutions twofold [53]. The wave-like pattern of mutations across the chromosome disappeared in bacteria growing on glucose minimal plates. However, the lower temperature retained the overall pattern, although the peaks of mutation rates that normally occurred 1000 kb from *oriC*, were shifted approximately 200 kb away, indicating that in addition to the growth rate of bacteria the growth medium composition affects the pattern of mutations across the chromosome [43].

There are also several other mechanisms that could be involved in the generation and maintenance of a wave-like pattern of mutations in the bacterial chromosome. Foster and colleagues [37] have shown that the density of mutations is greatest in the chromosomal regions predicted to have high superhelicity due to binding of nucleoid-associated proteins (NAPs) HU and Fis. The effect of NAPs on mutagenic and recombination processes will be discussed in more detail in the next section. The recent comprehensive study [43] suggests that the wave-like pattern of mutations across bacterial chromosome is the result of activity of several interconnected factors affecting DNA replication, repair, and chromosome. According to this study, while the transcription and the SOS response had only little effect, both the replication initiation, replication fork progression, and termination had significant effects. For example, when the origin of replication was relocated, the region of reduced mutation rates that surrounds the original *oriC* was re-established at the new origin location. Elimination of MapP protein that maintains the structure of the Ter macrodomain reduced mutation rates in the terminus region, indicating that highly structured DNA could be error prone. Niccum and colleagues also observed that both the proofreading activity and postreplicative error correction by MMR are effective close to the *oriC* region; these activities decline at one-third of the replichore then increase again, before declining again in the terminus region [43].

## 3. A Role of NAPs in Mutagenesis and Recombination Processes

NAPs play an important role in coordinating regulation of gene expression and nucleoid structure both at the local and global level [23]. Warnecke and colleagues [54] have analyzed the data of ChiP-seq experiments in *E. coli* where binding profiles of Fis, H-NS, and IHF were determined across the chromosome at different growth phases. They compared the NAP binding profiles with patterns of mutations across the genomes of 54 sequenced *E. coli* genomes. This study revealed weak but significant growth phase-specific effects of NAP occupancy on mutability [54]. In fact, in silico analysis of the NAP binding pattern during stationary phase revealed that bound regions experience reduced mutability for some mutation classes (e.g., C:G to T:A transitions). However, regions bound by NAPs during exponential phase exhibited higher mutability, suggesting that NAP binding enhances mutability by impeding DNA repair and/or interfering with DNA polymerase processivity during replication.

Recently developed chromosome conformation capture techniques confirmed that the *E. coli* chromosome is segmented into macrodomains and demonstrated that HU and Fis participate in the overall structuring of the chromosome, whereas H-NS affects short-range contacts by silencing DNA [12]. The result of this study also revealed that HU along with MukBEF is required to promote a megabase range of communications in the chromosome. Earlier studies disclosed a major role of the DNA binding protein MatP in organizing the terminus region of the chromosome into a Ter macrodomain and in restriction of MukBEF activity at the Ter domain surrounding the *ter* locus, thereby limiting the availability of topoisomerase IV at *ter* site and ensuring timely chromosome unlinking and segregation [55,56]. Lioy and colleagues demonstrated that HU and Fis partially counteract MatP’s restrictive effect on MukBEF activity and promote higher-order DNA organization [12]. As already mentioned above, most MA-WGS experiments and also several other studies suggested that the mutation rate is increased at the replication termination region [31,32,43]. Study of the involvement of NAPs in the generation of symmetrical wave-like patterns of mutations across the bacterial chromosome did not show significant correlations between the base substitutions patterns and published binding sites of the NAPs [43]. Nevertheless, HU and Fis had a role in establishing or maintaining the mutation rate pattern across the chromosome: the loss of HU changed the pattern and reduced the overall mutation rate, whereas the frequency of mutations was reduced in the terminus region similarly to that of the changes in the mutation rate pattern observed in the MatP-deficient bacteria; in the absence of Fis the overall mutation rate increased about twofold, but the wave pattern outside the *oriC* region was also flattened [37,43].

HU protein, which is one of the most abundant and conserved NAPs in eubacteria, also has a role in homologous recombination. HU sharply bends DNA and forms rigid nucleoprotein filaments at higher HU concentrations [57]. HU exists as a homodimer in most bacteria, but in enterobacteria HU can exist both in homodimeric forms HUα2 or HUβ2 and in heterodimeric form HUαβ [58]. In *E. coli*, the relative levels of these forms vary with growth phase of bacteria: while HUα2 is prevalent during exponential phase, HUαβ becomes prevailing in stationary phase or in other growth-restricting conditions [59]. The earlier studies with *E. coli* lacking both HU subunits revealed increased sensitivity of bacteria to γ-irradiation and demonstrated that HU protects DNA against γ-irradiation-induced breaks in vitro [60]. It was demonstrated that HU binds non-specifically to dsDNA, but it can also bind to bulged DNA, cruciform DNA, and single-strand breaks or gaps [61,62,63]. Genetic analyses of UV-irradiation-sensitive *E. coli* mutants indicated that HU has a role in homologous recombination [64]. Subsequent in vitro studies confirmed that HU can bind specifically to DNA recombination and repair intermediates [65].

HU and other NAPs have also been investigated for their role in the evolution of bacteria under stressful conditions. Under stressful, growth-restricting conditions, bacteria can rapidly evolve by a process known as adaptive, stress-induced or stationary-phase mutation [66,67]. The mechanisms of stationary-phase mutagenesis have been most frequently studied in *E. coli* strain FC40 carrying the *lac^–^* allele in F′ plasmid. The *E. coli* FC40 Lac system measures frameshift mutations that are dependent on mutagenic dsDNA break repair (MBR) and conjugal functions of F’ plasmid; this process involves participation of both the homologous recombination enzymes and translesion DNA polymerase Pol IV performing error-prone DNA synthesis [68,69,70,71,72,73]. Study by Williams and Foster [74] demonstrated that HU is involved in the occurrence of Lac^+^ revertants in *E. coli* FC40, possibly helping to channel recombination intermediates for resolution via a RuvABC-dependent mutagenic pathway instead a RecG-dependent non-mutagenic pathway. In the MBR examined in *E. coli* FC40 and its derivatives, only HU directly promotes homologous recombination, whereas other NAPs (Fis, H-NS, CspE, CspC, CbpA, Lrp, Hfq and IHF) participate in the regulation of mutagenic processes [75,76]. Using multiple in vivo tests, Moore and colleagues [77] observed that most studied NAPs promote MBR, but via different mechanisms [77]. The study by Moore and colleagues revealed that Fis is required for the activation of the SOS response leading to upregulation of the error-prone DNA polymerase Pol IV. H-NS facilitates MBR via transcriptional repression of superoxide dismutase gene *sodB* presumably by allowing the accumulation of reactive oxygen species (ROS); CspE participates in MBR via positive regulation of stationary phase sigma factor RpoS, and CbpA via promoting maintenance of F´ plasmid.

Among the studied NAPs, DNA binding protein from starved cells (Dps) has been the sole inhibitor of mutagenesis in the MBR pathway [75], and has had no effect on the pattern of mutation rate in the MA-WGS experiments [43]. Dps is the most abundant protein component of the nucleoid in stationary phase cells that compacts and co-crystalizes with DNA and protects DNA from reactive oxygen species [15]. The protective roles of Dps are achieved through a combination of nucleoid compaction, metal chelation, ferroxidase activity, and gene regulation [26]. The DNA-binding and ferroxidase activities of Dps are biochemically separable but function jointly to maintain DNA integrity and cellular viability [78]. The dramatic changes in the DNA topology induced by Dps have raised a question as to how DNA can be accessible to transcription and other processes connected with DNA metabolism in stationary phase cells. Recently, Janissen and colleagues [79] have demonstrated that RNA polymerase can freely enter and diffuse inside the Dps complexes, whereas other proteins such as restriction enzymes are blocked from accessing the DNA. The authors of this study have suggested that in such way Dps ensures transcriptional response to the encountered stress while protecting the genome from damage.

A role of NAPs on mutagenesis and recombination processes has been studied also in other bacteria, e.g., in *Bacillus subtilis* and *Pseudomonas putida*. Unlike *E. coli,* which contains several abundant nucleoid-associated proteins, e.g., HU, IHF, FIS, H-NS, StpA [23], *B. subtilis* contains only the homodimeric Hbsu protein, the counterpart of the *E. coli* HU, which is essential for cell viability [80], and another nucleoid-associated protein LrpC that stabilizes particular DNA structures [81]. Analysis of Hbsu mutations in *B. subtilis* in combination with mutations in different functions of homologous recombination in DNA damage tolerance and recombination assays demonstrated that Hbsu is required for DNA repair and recombination processes in this organism [82].

In *P. putida*, the homologous recombination events between a broad-host-range RK2-derived plasmid and the bacterial chromosome, restoring the expression of phenol monooxygenase gene *pheA,* are elevated during prolonged carbon starvation of bacteria in the presence of phenol, which is a potential carbon source and stressor for soil bacteria [83]. Both the usage of this assay and an assay detecting the homologous recombination events between the *pheA* alleles inserted randomly into different chromosomal loci of *P. putida* revealed that the chromosomal location of the homologous recombination target influences the frequency and dynamics of recombination events in *P. putida* [83,84]. A bioinformatic screen for sequence determinants affecting homologous recombination through the genome revealed that the chromosomal DNA regions of *P. putida* which flanked the test system in the strains exhibiting lower frequency of homologous recombination are strongly enriched in binding sites for at least three NAPs (FIS, IHF and MvaT/MvaU) when compared to those which expressed higher frequency of homologous recombination between plasmid and chromosome [83]. These results imply that DNA enriched with more NAPs could be less accessible to homologous recombination. Subsequent studies have focused on the elucidation of a role of IHF on *P. putida* genome maintenance. Among the abundant NAPs, IHF was initially identified as the protein essential for the site-specific integrative recombination of phage lambda in *E. coli* [85,86]. IHF affects many cellular functions including a variety of site-specific recombination events, transposition, chromosome replication initiation and gene expression [87]. In addition, IHF promotes site-specific integration of foreign DNA at the CRISPR locus [88]. Studies of homologous recombination and mutagenic processes in *P. putida* [89] revealed that both are facilitated by IHF in this organism. The positive effect of IHF on the frequency of homologous recombination varied depending on the chromosomal location of the recombination target and the type of the plasmid used in the assay. IHF had positive effect on the occurrence of point mutations in plasmidial assay, but in the chromosome IHF facilitated mutagenic processes only at certain target sequences or at certain chromosomal locations of these target sequences [89]. These results altogether suggest that the observed effects of IHF cannot be explained only by a role of this protein in the global regulation of transcription. It is possible that other mechanisms such as the accessibility of DNA to recombination and mutagenic processes could also be involved, but since the structural organization of DNA varies at different chromosomal positions, the requirement of IHF may also vary.

## 4. Concluding Remarks

**Figure 1:**
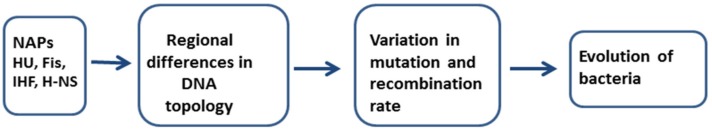
Involvement of nucleoid-associated proteins (NAPs) in mutability and recombination. Binding of NAPs (e.g., HU, Fis, IHF, H-NS) to the DNA alters its topology. Regional differences in the topology of chromosomal DNA may cause unequal access of chromosomal regions to mutagenic and recombination processes. Therefore, some regions in the genome have a potential to evolve faster than the others.

As already discussed above, multiple mechanisms may affect mutability and recombination events across the bacterial chromosome. The involvement of NAPs in both of these processes has become evident (Figure 1). Mutations can occur during the chromosome replication and as a result of the DNA repair synthesis at the sites of DNA damage. It is possible that regional differences in the topology of chromosomal DNA may cause unequal access of chromosomal regions to mutagenesis by influencing admission to DNA repair enzymes and involvement of specialized DNA polymerases in DNA repair synthesis. Thus, depending on the location of the potential target sequences in the chromosome, some mutational pathways may prevail over the others in the evolution of bacteria. Homologous recombination also has a great impact in bacterial evolution. In addition to inducing genetic rearrangements in bacteria (deletions, inversions, duplications in the chromosome), homologous recombination is an essential process for the integration of horizontally transferred genes into their new bacterial host genome [90]. While the different chromosomal loci are not equally accessible to homologous recombination, this may also affect the ability of the bacteria to evolve: depending on the location of the potential target sequences in the chromosome, some horizontally transferred DNA sequences could be more easily incorporated than others, which means that some regions in the genome may evolve faster than the others. For example, several studies have indicated that horizontally transferred DNA is clustered near the *terC* region in the bacterial genome, e.g., [91,92,93]. However, recent analysis of about 100 fully sequenced genomes of Rhodobacteraceae revealed some exceptions to this trend: prophages were identified closer to the origin of replication if conserved genes were clustered at the terminus, indicating that a model of preferential integration of transferred genes and bacteriophages at the terminus region might not be generalizable [94]. The effects of regional differences do not apply only to homologous recombination, but also to other recombination processes, such as the integration of prophages or transposition of mobile DNA elements. For example, Garcia-Russell and colleagues [95] observed unequal access of chromosomal regions for phage lambda integrase-mediated recombination between pairs of *att* sites at various loci in intrachromosomal recombination assay; this was also observed if one *att* site was located around the chromosome and a partner *att* site was present on RK2-derived plasmid. Also, the frequency of transposition of IS element IS*1411* into the *lacI* target gene vary at different chromosomal positions of *P. putida*, suggesting regional differences in DNA topology that may affect transposition [10]. It is important to bear in mind that the function of the foreign DNA sequence may also have an impact on its ability to integrate. A number of plasmids and other genetic elements carry genes for NAP orthologs that may affect NAP equilibrium and nucleoid structure in a host cell [21,96]. The upcoming years will possibly shed some light into the mechanisms of how the interactions between endogenous and foreign NAPs could affect the evolvability of the bacteria.
